# A Scoping Review of Measures Used to Assess Stress-Related Motor Dysfunction in Functional Movement Disorder

**DOI:** 10.5334/tohm.1042

**Published:** 2025-10-01

**Authors:** Chelsea Kinney, Mary Shotwell

**Affiliations:** 1 Rocky Mountain University of Health Professions, US

**Keywords:** Functional Neurological Disorder, Stress-related Motor Symptoms, Scoping Review

## Abstract

Functional Neurological Disorder (FND) is a complex condition characterized by a range of motor and non-motor symptoms, including tremors, dystonia, sensory disturbances and dissociative episodes. Functional Movement Disorder (FMD), a common motor subtype of FND, specifically involves abnormal involuntary movements without an identifiable structural or organic cause. There isn’t a validated assessment tool that currently exists to measure the specific impact of stress on motor symptom variability in FMD. The absence of such tools presents a barrier to accurate diagnosis, treatment planning, and longitudinal monitoring. This review focuses specifically on the FMD subtype, as it represents the most relevant phenotype for assessing stress-related motor dysfunction in the context of movement disorders. Following PRISMA-ScR guidelines, a systematic search was conducted across four major databases—PubMed, PsycINFO, Embase, and Scopus—using keywords related to FND, stress, motor symptoms, and psychometric evaluation. A total of 15 studies met inclusion criteria. Findings reveal a gap in available instruments: while several measures assess general stress (e.g., PSS-10, DASS-21) or motor symptoms (e.g., S-FMDRS), none are designed to capture the dynamic and stress-sensitive nature of motor dysfunction in FND. The review underscores the need for a patient-reported outcome (PRO) measure that is condition-specific, psychometrically validated, and capable of assessing real-time symptom variability linked to stress. Such a tool may offer significant benefits for clinical care and research by improving the precision of symptom monitoring, enhancing patient-provider communication, and guiding targeted interventions.

## Introduction

Functional Movement Disorders (FMDs) are neurological symptoms that affect movement but do not have a pathophysiological origin [1,27]. These manifestations often include tremors, limb weakness, dystonia and gait disturbances, which are frequently intensified by psychological stressors [9,25]. FMD symptoms often mimic conditions like Multiple Sclerosis and Parkinson’s disease, but standard diagnostic tests (e.g., EEGs, computed tomography scans [CT] magnetic resonance imaging [MRI]) often return normal or incidental findings that do not explain the patient’s symptoms. The estimated prevalence of FND is approximately 1.5% of the U.S. population, or roughly 5 million, accounting for up to 30% of cases seen in neurology clinics [14]. Prevalence studies outside the U.S. report similar trends, with FND affecting approximately 4–12 per 100,000 in the UK and parts of Europe [1,2]. Within the FND spectrum, FMDs specifically have an estimated incidence of 4 to 5 cases per 100,000 people each year and a prevalence of about 50 per 100,000, based on community registry data [11,29]. FMDs also make up approximately 3% to 8% of all visits to movement disorder clinics, further underscoring their clinical significance. Yet despite their reach, FND and FMD remain underdiagnosed, frequently misunderstood, and often stigmatized. The subjective nature of stress-related motor symptoms poses significant challenges in clinical assessment, necessitating a reliable psychometric tool to quantify these experiences.

One of the main challenges in managing FMDs lies in evaluating the variations in symptoms and severity, specifically the role of stress in exacerbating motor symptoms [8,9,27]. Stress is a common, though not exclusive, trigger; other contributors include pain, fatigue, and unknown or idiopathic factors. Clinicians rely heavily on generalized psychological inventories or subjective accounts that do not always capture fluctuations experienced in FMDs [3]. Some clinical signs such as the abductor sign, whack-a-mole sign, and swivel chair sign have been identified as useful positive indicators for diagnosing FMD, however, the lack of research presents a barrier to effective diagnosis, intervention, and monitoring in patients with FNDs [15,19].

## Background

Historically, clinicians have diagnosed FNDs through exclusion, ruling out neurological disorders rather than diagnosis based on positive signs. Today, there is a shift in clinical approaches towards the identification of “positive” signs for FMDs; including: Hoover’s sign (functional leg weakness), tremor variability, and symptoms that are improved with distraction [12,26]. The DSM-5 classifies FND under “Functional Neurological Symptom Disorder” and no longer requires the presence of a psychological stressor for diagnosis [23]. Research continues to highlight a strong link between stress, emotional dysregulation, and motor dysfunction in FMD patients.

A growing body of literature has investigated neurobiological pinnings of FNDs, which includes neurotransmitter dysregulation and altered brain connectivity. However, very few studies have specifically explored the mechanisms related to real-time stress-related symptom fluctuations. As a result, there is a pressing need for psychometrically sound instruments that can measure stress-related motor dysfunction from the patient’s perspective.

Patient-reported outcome (PRO) measures are increasingly used in clinical and research settings to capture subjective symptom experiences, quality of life, and treatment efficacy. A targeted PRO measure for FMDs, focused on stress-exacerbated symptom variability, might improve both intervention techniques and strategies [6,16,21]. Such a tool may allow clinicians to better understand the day-to-day symptom burden experienced by FMD patients and tailor treatments accordingly.

In contrast to the sparse psychometric toolkit available for FND, other conditions with overlapping biopsychosocial profiles—such as post-traumatic stress disorder (PTSD), chronic pain syndromes, and fibromyalgia—have established robust PRO measures that account for the influence of stress on symptomatology [9]. For instance, PTSD uses an assessment tool, known as the Clinician Administered PTSD Scale (CAPS), to measure stress-related symptom changes, allowing clinicians to assess symptom severity and triggers in a trauma-aware setting [31]. Chronic pain studies often use scales to evaluate how pain variability with emotional states and external stressors. These comparisons show how much the FND field lags in standardized patient assessments. Researchers and clinicians can learn from these conditions to serve as a methodological blueprint for future FND studies. Adopting similar multidimensional assessment strategies—including patient self-report, ecological momentary assessment (EMA), and physiologic biomarker integration—could vastly improve the sensitivity and clinical utility of FMD assessments.

Moreover, disorders like PTSD and chronic pain have benefitted from patient-centered research frameworks in which individuals contribute directly to tool development. This collaboration ensures that outcome measures reflect symptom dimensions that are meaningful to patients, not just those observable to clinicians or inferred from theory. FND research should follow this model by incorporating patient and clinician feedback into each stage of instrument design.

The purpose of this scoping review is to evaluate existing measures that may be applicable to stress-related motor symptom measurement in FMD, to identify gaps in the literature, and to provide a foundation for developing a validated PRO measure specifically designed for this population. This review aims to inform future research, improve clinical assessment practices, and ultimately contribute to better health outcomes for individuals living with FMD.

## Theoretical Frameworks

This review draws its theoretical foundation from the Stress-Vulnerability Model and the Dynamical Systems Theory. Originally used in psychiatric models to understand the onset of disorders like depression and schizophrenia, the Stress-Vulnerability Model proposes that those with inborn vulnerabilities may experience exacerbated symptoms when exposed to stressors, emphasizing the interaction between neurological motor symptoms in FMDs and stress [10]. If someone’s stress load surpasses their coping threshold, symptoms emerge or worsen. In the context of FMDs, this model posits the notion that individuals who are predisposed-either neurologically, psychologically or genetically-are more likely to develop motor symptoms when exposed to acute or chronic stressors. Vulnerabilities can include anxiety disorders, impaired stress regulation mechanisms within the brain or previous trauma. Though the DSM-5 no longer requires a psychological trigger for a diagnosis of FND, ongoing investigations suggest that perceived stress, even in the absence of psychological trauma, can provoke functional symptoms [7,24].

The Dynamical Systems Theory accentuates the multiplex interplay in biological, environmental and psychological factors dynamically influence symptom expression [5]. In FMD, the motor symptoms do not follow predictable neurological pathways, which supports the view that these symptoms result from dysfunctional interactions among multiple systems (e.g., attention, emotion regulation, motor control, and perception). The Dynamical Systems Theory aligns with the variating and context-dependent nature of FND symptoms, which range in frequency, severity and presentation based on emotional state, environmental triggers, and cognitive load [28]. It suggests that FND symptoms are emergent outcomes of complex, self-organizing processes that are sensitive to small perturbations—such as emotional stressors or physical exhaustion.

**Figure 1:**
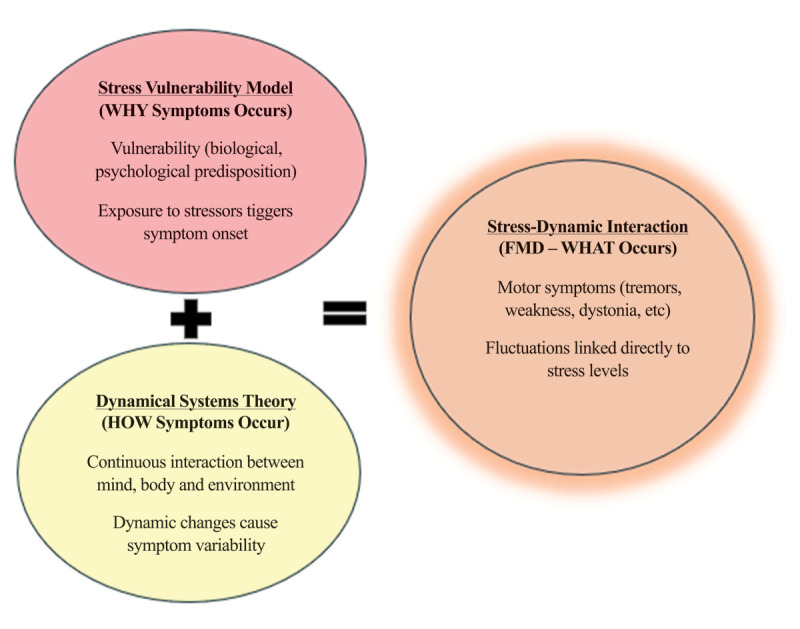
Vertical Model of Theoretical Frameworks Underpinning Symptom Expression in FND. This model was developed by the author to illustrate the conceptual relationship between stress vulnerability, dynamic symptom fluctuation, and functional neurological symptoms. Adapted and conceptualized by the author based on the Stress-Vulnerability Model [22] and Dynamical Systems Theory [5].

Figure 1 provides a diagram illustrating the theories that may interact as they apply to FND. This model underscores the necessity for a nuanced tool capable of capturing real-time symptom fluctuations driven by stress. The combination of the both models forms the “Stress-Dynamic Interaction Model”, which constructively links the “why” stress triggers motor symptoms (stress-vulnerability) with the “how” (dynamic symptoms fluctuations) these symptoms change in a complex, real-time manner. This model helps explain the initial emergence of symptoms in FNDs and their variability and sensitivity to stress over time. It also reflects the cyclical nature of the disorder, where stress-induced symptoms can, in turn, lead to more stress, perpetuating the condition in a feedback loop.

## Methods

### Search Strategy

A comprehensive literature search was conducted across multiple databases, including PubMed, PsycINFO, MEDLINE, and CINAHL. Key search terms included: “Functional Neurological Disorder,” “stress,” “motor symptoms,” “patient-reported outcomes,” and “psychometric evaluation.” Search filters (AND/OR) refined the search, and the reference lists of identified studies were screened for additional relevant sources. Inclusion criteria were original research, peer-reviewed articles, psychometric evaluation studies, and publications between 2010–2025. Only English-language, peer-reviewed full-text articles were included; non-English publications and conference abstracts were excluded due to feasibility constraints. Studies unrelated to stress-related motor symptoms or lacking psychometric validation details were excluded. Studies that included mixed populations with both functional and organic disorders were excluded unless outcomes for FND/FMD patients were reported separately. Full Boolean search strings, including field codes used for each database, are provided in Supplement 2 to support transparency and reproducibility.

### Screening & Selection Process

**Figure 2:**
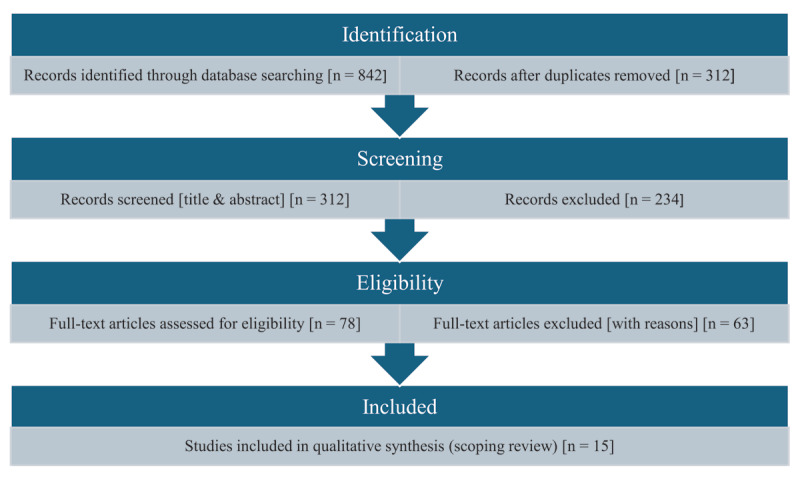
Prisma Flow Diagram. PRISMA-ScR flow diagram illustrating the screening and selection process. Adapted from Adapted from Tricco et al. [30].

Initial database searches yielded 842 articles. After removing duplicates, titles and abstracts of 312 articles were screened. Full-text review was conducted for 78 potentially relevant articles. A total of 63 articles were excluded following full-text review because they did not specifically examine stress-related motor symptoms or lacked psychometric evaluation data. Fifteen articles meeting inclusion criteria were included in this scoping review. A PRISMA-ScR flow diagram (see Figure 2) illustrates the detailed selection process.

### Data Extraction

Data were systematically extracted using a structured extraction form. Extracted information included author(s), year of publication, study objectives, participant characteristics, study design, psychometric properties (validity, reliability, responsiveness), and main outcomes related to FND stress-related motor symptoms. This review included only studies with participants who had a primary diagnosis of FND or FMD. Studies involving patients with both functional and organic movement disorders (e.g., Parkinson’s disease with functional overlay) were excluded unless the data specifically isolated outcomes for the FND/FMD subgroup. This distinction was made to maintain clarity in interpreting psychometric outcomes specific to the functional disorder population

### Data Synthesis and Analysis

Extracted data were narratively synthesized to describe constructs included on the measures, psychometric properties of the tools, and systematic presentation of findings. Comparative tables were created to facilitate interpretation of instrument quality, strengths, and limitations. Results highlight research gaps and inform recommendations for future PRO measure development tailored to FND stress-related motor symptoms. This review adhered to the PRISMA-ScR guidelines for scoping reviews. The completed PRISMA-ScR checklist is included in the supplementary materials (Supplement 1).

### Thematic Synthesis of Measures

A cross-study synthesis revealed three recurring themes in the design and use of instruments assessing stress-related motor symptoms in individuals with FND/FMD. First, there was a clear overreliance on general stress or mood inventories—such as the PSS-10, DASS-21, and HADS—that are not specifically tailored to the episodic or functional nature of motor symptom fluctuations. Second, several tools focused narrowly on motor severity, including the S-FMDRS and FNSD-RS, but offered limited insight into the interaction between stress exposure and symptom expression. Third, no existing tools effectively captured both domains (stress and motor dysfunction) in a single, integrated framework. This lack of integration likely limits their sensitivity and responsiveness to real-time symptom changes. Together, these themes highlight the need for future PRO instruments that blend psychological and neurological domains to reflect the lived experiences of FND/FMD patients.

## Results

### Overview of Identified Measures

This scoping review identified several instruments that have been used in evaluating motor dysfunction or stress in patient populations relevant to FMD. The key instruments identified include the Functional Neurological Symptom Disorder Rating Scale (FuNDRS), Simplified Functional Movement Disorder Rating Scale (S-FMDRS), Perceived Stress Scale (PSS-10), Depression Anxiety Stress Scales (DASS-21), and the Parkinson’s Disease Questionnaire (PDQ-39) (see Table 1). Each instrument provides unique insights, though none directly capture stress-induced symptom fluctuations specific to FMD:

- Functional Neurological Symptom Disorder Rating Scale (FuNDRS): Evaluates the severity of functional neurological symptoms without directly assessing stress as a contributing factor [3].
- Simplified Functional Movement Disorders Rating Scale (S-FMDRS): Assesses motor dysfunction in FMD but lacks sensitivity to stress-induced symptom fluctuations [18].
- Parkinson’s Disease Questionnaire (PDQ-39): A quality-of-life instrument originally developed for Parkinson’s disease populations, containing items related to mobility, activities of daily living, and emotional well-being. While not designed for FMD, it has occasionally been applied in movement disorder research due to symptom overlap [13,20].
- Perceived Stress Scale (PSS-10): Widely used to measure general stress levels; does not specifically address the impact of stress on motor function [4].
- Depression Anxiety Stress Scales-21 (DASS-21): Validated tool measuring stress, anxiety, and depression, but it lacks specificity for motor dysfunction in FMD [17].

**Table 1:** Measures related to Stress-Related Motor Symptoms in Functional Neurological Disorder (FND).


INSTRUMENT NAME	AUTHOR & YEAR	OVERVIEW	POPULATION	RELIABILITY	VALIDITY TYPES	RESPONSIVENESS	STRENGTHS	LIMITATIONS

FuNDRS	Brar [3]	Assesses symptom severity and frequency specifically in FND patients.	FND patients	α = 0.78	Construct validity	Moderate	Easy administration	Not explicitly stress-focused

S-FMDRS	Nielsen et al. [18]	Simplified clinician-administered scale to evaluate motor symptoms in functional movement disorders.	Functional Movement Disorder	ICC = 0.85	Criterion, Construct	Good	Clinician-friendly	Limited stress-related questions

PSS-10	Cohen et al. [4]	Widely used scale measuring perceived stress in various populations.	General populations, patients with stress	α = 0.89	Construct, Criterion	High	Widely validated, stress-specific	Not specific to motor symptoms

DASS-21	Lovibond & Lovibond [17]	Measures depression, anxiety, and stress in general populations.	Psychological distress	α = 0.94	Construct validity	High	Well-validated, comprehensive	General psychological distress, lacks motor focus

PDQ-39	Peto et al. [20]	Measures quality of life and symptom burden specifically in Parkinson’s disease.	Parkinson’s patients	α = 0.93	Construct, Criterion	High	Validated motor symptom assessment	Disease-specific (Parkinson’s), not stress-focused


### Reliability and Validity Analysis

After reviewing the PRO measures, it is clear that the reliability and validity of the PROs’ psychometric properties vary significantly. For the reliability metrics, Cronbach’s alpha and Intraclass Correlation Coefficient (ICC) typically display good stability and internal consistency [29]. For example, The DASS-21 demonstrated strong internal consistency (Cronbach’s α = 0.94; [17,29]. The S-FMDRS presents an ICC of 0.85, showing robust inter-rater reliability across clinician ratings. However, reliability alone does not warrant a tool’s suitability for capturing stress-induced motor dysfunction.

The validity of the measures in this review shows great variety, specifically regarding construct and content validity. For example, the PSS-10 is extensively validated to evaluate general stress but lacks assessment of motor function, making it questionable for FMD-specific research. Similarly, the PDQ-39 assesses quality of life in Parkinson’s disease populations, including domains such as mobility, daily activities, and emotional well-being. However, it does not evaluate stress-related motor symptom fluctuations, which limits its relevance for FMD populations.

### Summary of Findings

This scoping review revealed several key trends in the measures that might be used for FMD evaluations. A consistent pattern was the frequent use of general psychological instruments rather than tools explicitly designed for FMDs. The review shows an apparent conflict between existing assessments and complex clinical realities faced by FMD patients. Another notable finding was the heavy reliance on instruments initially developed for other conditions—for instance, the PDQ-39, which was designed for Parkinson’s disease, or the DASS-21, which targets general anxiety and depression. While these tools may offer some insight, their relevance to FMD may be limited, as the measures may overlook the disorder’s unique symptom presentation and underlying mechanisms.

Although many of the reviewed instruments show acceptable reliability and internal consistency levels, they do not seem to account for the nuanced relationship between stress and motor symptom variability which commonly observed in FMD patients. In fact, none of the selected measures mention real-time or short-term fluctuations in FMD symptoms, which is an essential component for effective clinical monitoring and a timely therapeutic response. While the instruments may be psychometrically sound in their original contexts, they fall short when applied to FMD. These gaps highlight the pressing need for a dedicated, condition-specific patient-reported outcome (PRO) measure that may more accurately reflect the lived experience of stress-related motor dysfunction in FND.

### Measurement Gaps & Trends

There were several measurement gaps identified through this scoping review, primarily relating to the absence of specialized tools designed explicitly for assessing stress-induced motor fluctuations in FMD patients. Existing tools either measure general stress levels or motor symptom severity independently without adequately capturing the dynamic interplay between these two critical dimensions. Another critical gap is the absence of measures sensitive enough to detect short-term symptom fluctuations triggered by everyday stressors, a common phenomenon in FMD clinical presentations. The absence of longitudinal data across many existing scales presents another critical limitation, calling into question their potential utility in clinical settings. Without evidence that these tools can reliably track symptom changes over time, clinicians are left without clear guidance for evaluating treatment progress or adjusting care plans.

Moreover, most of these measures were developed with little to no input from patients, perhaps indicating they may miss vital aspects of the FMD experience that matter most to affected patients. This lack of patient-centered design raises concerns about whether current tools truly capture the full range of symptom variability—particularly stress-induced fluctuations that are often central to the clinical picture in FMD. There is a clear necessity for a validated, patient-informed PRO measure specifically designed to assess stress-related symptom variability. Developing such a tool would not only enhance clinical assessment but also support more tailored and effective interventions for individuals with FMD. These findings highlight consistent patterns in the available instruments and underscore several gaps that warrant further analysis and interpretation.

## Discussion

### Key Findings

This scoping review identifies a gap in the landscape of FMDs-particularly the lack of psychometrically validated tools created to evaluate the interaction between motor symptom variability and stress. Although existing tools (FNSD-RS, S-FMDRS and PSS-10) offer a general perception into perceived stress or symptom severity, there is not one PRO measure that adequately captures the dynamic interaction between motor symptoms and stress that reflects the lived experiences of FMD patients. The absence of said measure presents a crucial barrier to progress in both research and clinical care.

Findings indicate that most instruments used with the FMD populations were either designed to measure broader psychological stress or were adapted from unrelated conditions. As a result, they fail to offer the specificity needed for clinicians to understand how stress impacts real-time symptom expression. Though instruments like the PDQ-39 and DASS-21 are proven to be reliable and validating for assessing psychomatic symptoms or stress in prevalent populations, they lack the depth, focus and precision on motor dysfunction for a comprehensive FMD evaluation. This highlights a serious need for targeted tools that integrate both physiological and psychological symptom domains specific to FMD.

Moreover, the current tools demonstrate limited responsiveness to symptom fluctuation, an essential feature for monitoring treatment efficacy and disease progression in a disorder as variable as FMD. Many patients with FMD report episodic symptom intensification related to stress exposure, but without tools that reflect these real-time experiences, clinical evaluation remains incomplete and often misleading. The development of a validated, condition-specific patient-reported outcome (PRO) measure is not just a theoretical recommendation—it is a clinical imperative.

### Limitations

This review is not without limitations. The most significant drawback was the limited number of studies focused on evaluation of stress-related motor symptoms in FMDs. While studies on general stress and FNDs were common, very few presented rigorous psychometric data or validated tools. The limited data significantly constrained efforts to generalize findings or carry out meta-analytical syntheses, especially when comparing diverse FND subtypes. In addition, well-established general measures such as the SF-36, PHQ-9, GAD-7, and HADS were not part of the initial search strategy, as the scope was narrowed to tools with specific motor or FND-related applications. These tools may offer complementary insights and should be considered in future reviews.

Another key limitation is the heterogeneity of study designs and inconsistent outcome measures used across the reviewed literature. The lack of standardization makes comparing results challenging or determining which instruments perform best across settings and populations. Many studies rely heavily on cross-sectional data, which restricts the ability to assess predictive validity, test-retest reliability, or longitudinal symptom patterns—critical psychometric features for any clinical tool.

The sample size was another recurrent limitation. Studies such as those by Bennett et al. and Espay et al. often used small or narrowly defined samples, reducing statistical power and generalizability. In addition, these smaller studies also lacked diversity within participant demographics, raising concerns about how well the findings apply to real-world patients with FNDs.

Few studies clearly distinguished between different FND subtypes (e.g., functional tremor vs. functional weakness), which limits understanding of whether different symptom profiles require separate measurement strategies. Subtype-specific analysis could significantly improve the specificity and sensitivity of PRO measures, making this a key area for future investigation.

Potential reporting bias remains a concern with patient-reported outcomes. Psychological state, memory recall, and symptom interpretation can all influence how patients respond to questionnaires. While this limitation is inherent to PRO measures in general, it is particularly relevant in FND, where emotional dysregulation and stress reactivity are core features. Future measures should include built-in mechanisms to reduce this bias—such as structured response formats, anchored rating scales, and possibly even physiological correlates.

Finally, though this review did not stratify psychometric outcomes by geographic region or ethnicity, future research should explore whether symptom manifestation and response patterns differ across cultural groups. Variations in healthcare access, stigma, and explanatory models may influence how stress-related motor symptoms are perceived, reported, or measured.

### Future Directions

The findings from this scoping review highlight several opportunities for future research in the assessment and clinical management of FNDs. First and foremost, the development of a novel, psychometrically validated PRO measure that specifically assesses stress-related motor symptom variability in individuals with FMD should be a primary focus. This measure would not only fill a critical gap identified in the literature but also provide a much-needed tool to improve patient-clinician communication, enhance diagnostic clarity, and support more individualized treatment planning.

Following the development and initial validation of the PRO, future research should focus on evaluating its longitudinal utility. Long-term studies could assess the PRO’s sensitivity to changes over time, particularly in response to interventions such as cognitive behavioral therapy (CBT), physical therapy, or stress-reduction programs. These studies would help establish the tool’s responsiveness and predictive validity, which are essential for determining its real-world clinical value.

In addition, future studies could explore cross-cultural validation and translation of the instrument to increase its global applicability. FMD affects individuals from a wide range of backgrounds, and cultural differences in stress perception, symptom reporting, and healthcare access may influence how patients respond to such a tool. Expanding the measure’s usability across diverse populations would enhance its relevance in international clinical and research settings. Another avenue for exploration involves integrating the PRO measure into digital health platforms. Incorporating the tool into mobile apps or electronic health records (EHRs) could support remote symptom tracking and provide real-time feedback to both patients and healthcare providers. This integration could significantly enhance the management of FMD by allowing more dynamic, continuous monitoring outside the clinical setting.

Finally, future research might also consider the neurobiological correlates of stress-related symptom fluctuations in FMD. For example, studies might examine whether scores from the new PRO correlate with biomarkers such as cortisol levels, heart rate variability, or functional neuroimaging data. Linking self-reported symptom variability with physiological data would strengthen the scientific basis of the tool and deepen our understanding of FND’s underlying mechanisms.

## Conclusion

This scoping review emphasizing the demand for specialized measures to assess stress-related motor dysfunction in FMD. Addressing this lack of research will guide therapeutic interventions, enhance diagnostic precision, guide therapeutic interventions, and ultimately improve the quality of life for individuals affected by this complex disorder. The findings highlight that while various measures exist, none comprehensively capture the interplay between stress and motor dysfunction. There is a significant opportunity for researchers and clinicians to collaborate in developing and validating new PRO measures tailored to the unique symptom patterns of FMD. Additionally, improving awareness and education about FMD-specific assessment tools could help standardize clinical evaluations and improve treatment outcomes. Future advancements in digital health, neurophysiological monitoring and psychometric research may lead to a more refined understanding of stress-related motor dysfunction, ultimately benefiting both patients and healthcare providers.

## Additional Files

The additional files for this article can be found as follows:

10.5334/tohm.1042.s1 Supplement 1. Preferred Reporting Items for Systematic reviews and Meta-Analyses extension for Scoping Reviews (PRISMA-ScR) Checklist.

10.5334/tohm.1042.s2 Supplement 2. Full Search Strategy.
