# Supplementary material for: A Scoping Review of Measures Used to Assess Stress-Related Motor Dysfunction in Functional Movement Disorder
Source: Tremor Other Hyperkinet Mov (N Y). 2025 Oct 1;15:49. doi: 10.5334/tohm.1042 (PMC12493031; doi:10.5334/tohm.1042)
Supplement: Supplement 2. — Full Search Strategy. [file tohm-15-1-1042-s2.pdf]

Supplement 2: Full Search Strategy

Database: PubMed
Search String: ("functional neurological disorder" OR "functional movement disorder"[OR FND OR FMD AND ("stress" OR "psychological stress"[MeSH Terms]) AND ("motor symptoms" OR "movement disorder"[tiab] OR "motor function"

Filters Applied: English, Humans, 2010–2025

Database: PsycINFO
Search String: ("functional neurological disorder" OR "functional movement disorder" OR FND OR FMD) AND (stress OR psychological stress) AND (motor symptoms OR movement disorder OR motor function)

Filters Applied: Peer-reviewed, English, 2010–2025

Database: MEDLINE
Search String: ("functional neurological disorder" OR "functional movement disorder" OR FND OR FMD) AND ("motor symptoms" OR "movement disorder") AND (stress OR "psychological stress")

Filters Applied: English, 2010–2025

Database: CINAHL
Search String: ("functional neurological disorder" OR "functional movement disorder") AND (stress OR psychological stress) AND (motor symptoms OR motor function OR movement disorder)

Filters Applied: English, Peer-reviewed, 2010–2025
